# Inflammaging Beyond Biomarkers: Molecular Mechanisms and Therapeutic Opportunities

**DOI:** 10.3390/cimb48060629

**Published:** 2026-06-16

**Authors:** Amelia Tero-Vescan, Ruxandra Ștefănescu, Amalia Pușcaș, Mădălina Buț, Bianca-Eugenia Ősz, Mark Slevin

**Affiliations:** 1 Biochemistry Department, Faculty of Medicine in English, George Emil Palade University of Medicine, Pharmacy, Science, and Technology of Târgu Mures, 540142 Târgu Mures, Romania; amelia.tero-vescan@umfst.ro (A.T.-V.); madalina.but@umfst.ro (M.B.); 2 Pharmacognosy and Phytotherapy Department, Faculty of Pharmacy, George Emil Palade University of Medicine, Pharmacy, Science, and Technology of Târgu Mures, 540142 Târgu Mures, Romania; 3 Biochemistry Department, Faculty of Pharmacy, George Emil Palade University of Medicine, Pharmacy, Science, and Technology of Târgu Mures, 540142 Târgu Mures, Romania; amalia.puscas@umfst.ro; 4 Pharmacology and Clinical Pharmacy Department, Faculty of Pharmacy, George Emil Palade University of Medicine, Pharmacy, Science, and Technology of Târgu Mures, 540142 Târgu Mures, Romania; bianca.osz@umfst.ro; 5 Center for Advanced Medical and Pharmaceutical Research, George Emil Palade University of Medicine, Pharmacy, Science, and Technology of Târgu Mures, 540142 Târgu Mures, Romania; mark.slevin@umfst.ro

**Keywords:** inflammaging, cellular senescence, SASP, NF-κB, NLRP3 inflammasome, cGAS–STING, JAK/STAT signaling, p38 MAPK, endothelial dysfunction, adipose tissue inflammation

## Abstract

Inflammaging is defined as chronic low-grade inflammation associated with aging and is increasingly recognized as a dynamic and mechanistically driven biological process rather than a state adequately described by circulating biomarkers alone. Traditional inflammatory markers alone, including interleukin-6 (IL-6), tumor necrosis factor-alpha (TNF-α), and C-reactive Protein (CRP), fail to capture the complexity, tissue specificity, and causal architecture of inflammaging. Recent experimental evidence has demonstrated that diverse upstream drivers, including immunosenescence, gut microbiome dysbiosis, metabolic dysfunction, and cellular senescence, converge on a limited number of central inflammatory hubs, including nuclear factor kappa-light-chain-enhancer of activated B cells (NF-κB), NOD-like receptor family pyrin domain containing 3 (NLRP3) inflammasome, GMP–AMP synthase–stimulator of interferon genes (cGAS–STING), Janus kinase/signal transducer and activator of transcription (JAK/STAT), and p38 mitogen-activated protein kinase (p38 MAPK) signaling. These mechanistic nodes represent promising therapeutic targets, potentially modifiable biological processes, and support the emerging concept of ‘druggable inflammaging’, whereby senotherapeutics, inflammasome inhibitors, innate immune modulators, and metabolic interventions may actively modify aging-associated inflammatory biology rather than simply monitor it through biomarkers. This review highlights a paradigm shift from biomarker-based assessment toward mechanism-based intervention, where inflammaging can be characterized as a modifiable biological process and a central target for precision pharmacological strategies in aging-related diseases.

## 1. Introduction

Aging is no longer perceived as a passive process of decline but rather as an active, biologically regulated process involving immune and metabolic mechanisms that lead to the establishment of a chronic, low-grade inflammatory state known as inflammaging. The causes of inflammaging are multiple and convergent and involve sterile immune activation mechanisms, including cellular senescence and the senescence-associated secretory phenotype (SASP), mitochondrial dysfunction and the release of damage-associated molecular patterns (DAMPs), dysregulated proteostasis, impaired gut barrier function, and age-related alterations in innate and adaptive immunity [1]. This persistent elevation of inflammatory tone amplifies vulnerability to cardiometabolic diseases, neurodegeneration, frailty, and impaired tissue regeneration [2].

Inflammatory biomarkers such as cytokines, high-sensitivity C-reactive protein (hsCRP), interleukin-6 (IL-6), interleukin-1β (IL-1β), and tumor necrosis factor-α (TNF-α) do not adequately reflect the complexity and causality of inflammaging. Moreover, when these markers are interpreted outside their biological context, they may provide misleading information. IL-6 is classically regarded as a marker of inflammation; however, it is actively released from skeletal muscle during physical exercise, where it functions positively as an anti-inflammatory, insulin-sensitizing myokine with metabolic effects, including the induction of IL-10 and the inhibition of TNF-α production [3,4].

This limitation is also observed for other inflammatory markers. IL-1β signaling often fails to distinguish adaptive from maladaptive inflammatory states [5]. Leptin, which is commonly regarded as a proinflammatory adipokine in obesity and aging, is indispensable for immune competence, energy homeostasis, and tissue repair [6], whilst TNF-α, typically associated with catabolic and proinflammatory signaling, also participates in tightly regulated processes of immune surveillance and tissue remodeling [7]. Finally, CRP reflects the presence of inflammation but provides limited insight into its origin, cellular drivers, or biological consequences [8].

Taking all these considerations into account, a central translational challenge is to move from measuring inflammaging to actively modulating it, treating inflammaging as a dynamic and targetable biological process rather than as a static, biomarker-defined phenotype.

Inflammaging should not be viewed as a diffuse systemic phenomenon but rather as a network of discrete, actionable biological nodes with defined upstream triggers, downstream effectors, and context-dependent tissue outcomes. For example, studies in naturally aged mice have shown that maladaptive activation of cyclic GMP–AMP synthase (cGAS)–stimulator of interferon genes (STING) signaling in brain-resident immune cells induces a type I interferon response that is associated with neuronal loss and cognitive impairment, indicating that cytosolic nucleic acid sensing could actively drive inflammaging rather than merely reflecting it [9]. In parallel, other experimental studies on mice using high-resolution, multitissue mapping approaches have demonstrated that cellular senescence is not a single uniform program but instead represents a heterogeneous ecosystem capable of creating an “aged-like inflamed niche” that restricts stem-cell function and impairs tissue regeneration [10].

Based on the existing experimental literature evidence, this review aims to introduce the concept of “druggable inflammaging,” with the objective of connecting key mechanistic nodes such as the SASP, cGAS–STING signaling, and inflammasome IL-1β/IL-6 axes to candidate therapeutic strategies. These strategies include the elimination or reprogramming of proinflammatory senescent cells, the inhibition of innate immune inflammasome and cytokine circuits, the interception of nucleic acid sensing and interferon signaling programs, and the restoration of metabolic–immune homeostasis.

## 2. Methodology

For this narrative review, a structured literature search was performed using the PubMed and Scopus databases. The search strategy combined keywords related to inflammaging and its principal mechanistic drivers and therapeutic targets, including: “inflammaging”, “cellular senescence”, “senescence-associated secretory phenotype (SASP)”, “NF-κB”, “NLRP3 inflammasome”, “cGAS–STING”, “JAK/STAT signaling”, “p38 MAPK”, “immunosenescence”, “gut microbiome”, “metabolic dysfunction”, “senolytics”, “senomorphics”, and “anti-inflammatory therapies”. The literature search was last updated in January 2026. The initial search across PubMed and Scopus identified approximately 420 records. After screening titles and abstracts for relevance and applying the predefined eligibility criteria, approximately 160 articles were selected for full-text assessment. Following evaluation of their scientific relevance and contribution to the scope of the review, 100 publications were ultimately included in the final narrative synthesis.

Articles were screened based on their title, abstract, and relevance to the scope of the review. Non-English publications, conference abstracts, editorials, and gray literature were excluded. Studies that did not address the molecular mechanisms, biological drivers, or therapeutic modulation of inflammaging were also excluded.

Both original research articles and review papers were considered, including preclinical, translational, and clinical studies. Particular emphasis was placed on recent publications and experimental evidence supporting the concept of “druggable inflammaging”, focusing on molecular signaling pathways and pharmacological interventions capable of modulating chronic low-grade inflammation associated with aging. This approach aimed to provide a comprehensive and up-to-date synthesis of current knowledge regarding the mechanistic basis and therapeutic targeting of inflammaging.

## 3. Identifying the Primary Sources of Chronic Low-Grade Inflammation

Chronic low-grade inflammation during aging arises from multiple primary biological sources, including immune system remodeling, altered host–microbiome interactions, and age-related metabolic and tissue-specific changes, which together establish a persistent proinflammatory milieu characteristic of inflammaging.

The paradox of immunosenescence, which is a central contributor to inflammaging, refers to the coexistence of two seemingly opposite processes: age-dependent immune dysfunction characterized by reduced T-cell diversity and impaired effector function, alongside a state of chronic low-grade inflammation driven by the expansion of proinflammatory immune cell subsets and cytokine production, as stated by Fulop et al. [11]. Experimental single-cell, multiorgan immune profiling in naturally aged mice revealed extensive age-related immune remodeling across tissues, characterized by a reduction in adaptive immune mechanisms particularly CD4-positive T lymphocytes (CD4^+^ T cells), CD8-positive T lymphocytes (CD8^+^ T cells), natural killer (NK) cells and plasmacytoid dendritic cells (pDCs), expansion of myeloid lineages (monocytes and granulocytes), and phenotypic shifts consistent with increased inflammatory tone through increased major histocompatibility complex class II (MHC class II) expression on macrophages and loss of cells with antiviral and clearance functions. These findings support the concept of immunosenescence as an active driver, rather than a passive consequence, of inflammaging [12].

Further studies in aged mice also revealed that age-associated gut dysbiosis was accompanied by signs of intestinal barrier disruption and increased systemic exposure to gut-derived pathogen-associated molecular patterns, as reflected by elevated levels of circulating lipopolysaccharide (LPS) binding protein and enhanced toll-like receptor 4 (TLR4) signaling [13]. Notably, transplantation of microbiota from aged donors into young germ-free mice was sufficient to induce colonic inflammatory gene expression and systemic markers consistent with innate immune activation, providing direct experimental evidence for a causal role of the gut microbiome in driving inflammaging [13]. Human cohort studies have similarly demonstrated extensive age-associated remodeling of the gut microbiome and circulating metabolome in nonagenarians and centenarians, together with changes in inflammation-associated metabolites and immune-related pathways, consistent with altered host–microbiome interactions and phenotypical switching to low-grade inflammation during aging [14].

This phenomenon was confirmed by Khosla et al., who introduced and reviewed the ‘geroscience hypothesis’ and role of cellular senescence in several endocrine diseases, including osteoporosis, metabolic syndrome, and type 2 diabetes mellitus, as well as other endocrine conditions, describe how metabolic inflammation represented a major source of chronic low-grade inflammatory signaling during aging, particularly through age-related remodeling of adipose tissue and ectopic lipid accumulation [15].

In support of this theory, experimental studies in C57BL/6 mice demonstrated that aging in animals fed Ad Libitum resulted in excessive ectopic lipid accumulation in nonadipose tissues and particularly in the liver, whilst mice subjected to dietary restriction did not. Genetic (via suicide gene-mediated ablation of p16Ink4a-expressing senescent cells in INK-ATTAC mice) or pharmacological clearance of senescent cells (by administration of dasatinib plus quercetin) was sufficient to reduce hepatic steatosis and inflammatory immune infiltration [16].

**Figure 1:**
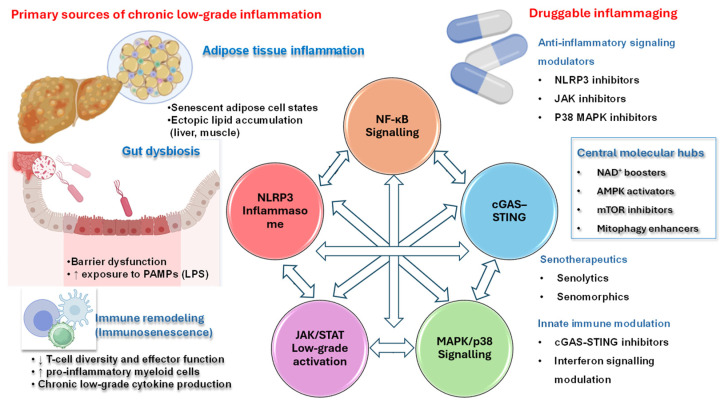
Primary sources of chronic low-grade inflammation converge on central molecular hubs that orchestrate inflammaging. Immune remodeling associated with immunosenescence, altered host–microbiome interactions, and metabolic tissue dysfunction act as upstream drivers of persistent inflammatory signaling. These inputs are integrated through a limited number of central molecular hubs, including the NF-κB, NLRP3 inflammasome, cGAS–STING, JAK/STAT, and p38 MAPK pathways. Targeting these hubs through senotherapeutics, innate immune modulators, and metabolic interventions represents a framework for druggable inflammaging. Abbreviations: LPS—lipopolysaccharide, PAMPs—pathogen-associated molecular patterns, NF-κB—nuclear factor kappa B, NLRP3—NOD-like receptor family pyrin domain containing 3, cGAS—cyclic GMP–AMP synthase, STING—stimulator of interferon genes, JAK—Janus kinase, STAT—signal transducer and activator of transcription, p38 MAPK—p38 mitogen-activated protein kinase, AMPK—AMP-activated protein kinase, and mTOR—mechanistic target of rapamycin, ↑ up arrow means increase, ↓ down arrow means decrease. Created in BioRender. Tero-Vescan, A. (2026) https://BioRender.com/k3rsmqg (accessed on 10 June 2026).

This work and that of others strongly support the hypothesis that cellular senescence drives mitochondrial dysfunction, impaired fatty acid oxidation, and proinflammatory signaling whilst establishing a causal link between senescence, ectopic lipid deposition, and inflammaging in the aging liver. Similarly, the data demonstrate that chronic low-grade inflammation in aging does not arise from a single source, but rather from the convergence of several factors that establish a persistent inflammatory milieu. In addition to these upstream sources, the inflammatory phenotype of aging is directed through a limited number of central molecular hubs, which integrate diverse signals into maladaptive immune and metabolic responses and are discussed in the following section. Figure 1 provides an overview of the major primary sources of low-grade inflammation, their convergence toward central mechanistic hubs, and potential therapeutic intervention points.

## 4. Convergent Molecular Signaling Hubs Integrate Inflammaging Pathogenesis and Represent Potentially Druggable Nodes

A defining feature of inflammaging is that highly diverse upstream drivers (immunosenescence, microbiome-derived PAMPs, metabolic tissue dysfunction and senescence/SASP) converge onto a relatively small number of intracellular signaling hubs. These hubs act as signal integrators that translate danger sensing, cytokine cues, and metabolic stress into persistent inflammatory gene programs and maladaptive tissue responses and are considered attractive candidates for the concept of “druggable inflammaging.”

Mechanistically, low-grade inflammation in aging arises from persistent, submaximal activation of inflammatory signaling pathways rather than from acute immune responses. Age-associated mitochondrial dysfunction, accumulation of senescent cells, chronic exposure to low levels of microbial and endogenous danger signals, and impaired inflammatory resolution converge on sustained inhibitor of κB kinase (IKK)–NF-κB activation. This tonic NF-κB signaling drives continuous, low-level expression of inflammatory mediators and SASP components, creating a self-amplifying inflammatory milieu that cannot trigger tissue repair or immune clearance but is sufficient to promote chronic tissue dysfunction, as shown in various in vivo studies, for example, in growth hormone receptor knockout mice [17], and pregnancy-associated plasma protein A knockout mice [18].

Numerous experimental studies have focused on the “druggable inflammaging” paradigm in cardiovascular disease, demonstrating that senescence and immune-driven inflammatory hubs (e.g., p16^INK4a^–STAT3–NLRP3 and cGAS–STING) can be therapeutically targeted thereby providing a proof-of-concept. These strategies include the effective use of senolytics such as the dasatinib + quercetin combination for the repair of myocardial tissue from C57BL/6J female mice with myocardial infarction [19]. Inflammasome-directed approaches targeting the p16INK4a–STAT3–NLRP3 axis, including senomorphic nanotherapeutic strategies and cardiomyocyte-specific p16INK4a knockout models, have been shown to attenuate STAT3/NLRP3-driven inflammaging and tissue dysfunction. [20], and pathway-specific inhibitors such as pharmacological inhibition of the NLRP3 inflammasome with MCC950, which attenuated metabolic dysfunction concomitant with increased autophagy and inhibition of the mTOR pathway in aged mice [21]. Phenotypically, these interventions clearly reduce inflammation and fibrosis in these models, protecting against adverse cellular and tissue remodelling.

### 4.1. NF-κB as a Central Integrator and Druggable Node in Inflammaging

**Figure 2:**
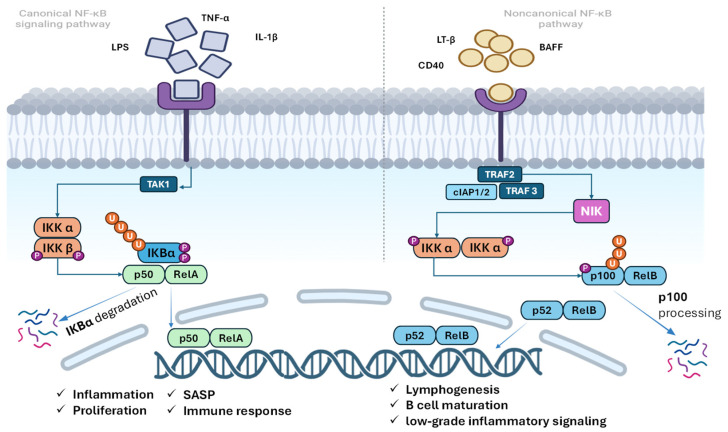
Canonical and noncanonical NF-κB signaling pathways involved in inflammaging. Canonical NF-κB signaling (**left**) is activated by inflammatory stimuli such as LPS, TNF-α, and IL-1β, which act on specific cytokine receptors and signal through TAK1. This leads to the activation of the IKK complex (IKKα, IKKβ, and NEMO), the phosphorylation and ubiquitin-mediated degradation of IκBα, and the nuclear translocation of p50/RelA (p65) heterodimers, which drive the transcription of proinflammatory cytokines, chemokines, and SASP components. In parallel, the noncanonical NF-κB pathway (**right**) is activated by specific members of the TNF receptor superfamily, including LT-β, CD40, and BAFF. This pathway leads to stabilization of NIK and subsequent activation of IKKα homodimers, resulting in phosphorylation and proteolytic processing of p100 to p52. The p52/RelB complex translocates to the nucleus and regulates genes involved in immune cell differentiation, lymphoid tissue organization, and sustained low-grade inflammatory signaling. Together, the canonical and noncanonical NF-κB pathways integrate immune, metabolic, and stress-related signals to establish and maintain the chronic low-grade inflammatory milieu characteristic of inflammaging, thereby contributing to tissue dysfunction and age-associated disease susceptibility. Abreviations: LPS—lipopolysaccharide, TNF-α—tumor necrosis factor-α, IL-1β—interleukin-1β, TAK1—TGF-β–activated kinase 1, IKK—inhibitor of κB kinase, NF-κB—nuclear factor kappa B, BAFF—B-cell activating factor, SASP—senescence-associated secretory phenotype, LT-β—lymphotoxin-β, NIK—NF-κB-inducing kinase. Created in BioRender. Tero-Vescan, A. (2026) https://BioRender.com/wrct50g (accessed on 10 June 2026).

NF-κB is a key central link between the initiation and maintenance of the inflammatory tone. Different upstream inflammatory triggers, such as Toll-like receptor (TLR) ligands derived from the microbiota, proinflammatory cytokines (e.g., TNF-α and IL-1β), oxidative stress, mitochondrial dysfunction, and DNA damage responses, converge on the IKK–NF-κB axis, leading to IκBα phosphorylation and degradation, activation of p65, and induction of inflammatory cytokine production, key components of the SASP, as shown in Figure 2.

The activation of the IKK–NF-κB axis by LPS (100 µg mL^−1^) or TNFα (50 ng mL^−1^) has been demonstrated in vitro in human proximal tubular epithelial HK-2 cells [22], whilst a comprehensive translational study integrating human clinical data, in vivo murine models, and in vitro endothelial systems demonstrated that the activation of the RIPK1–IKK–NF-κB axis was a key driver of endothelial dysfunction in chronic kidney disease (CKD). Mechanistically, elevated RIPK1 expression in abdominal aortic endothelial cells was associated with ER stress, reduced eNOS expression, increased ICAM-1 levels, and sustained NF-κB–mediated inflammation [23].

In support of these findings, Zhang et al. demonstrated that pharmacological inhibition of IKK-mediated NF-κB activation via the small molecule SR12343 in an Ercc1^−^ progeroid murine model of accelerated aging, significantly reduced the expression of markers of cellular senescence and SASP, decreased inflammatory signaling, improved health span readout, including muscle fiber integrity and muscle strength; and reduced overall frailty. In vitro studies from the same authors showed a significant reduction in key SASP-related and inflammatory markers, including IL-6, IL-1β, TNF-α, p16^Ink4a^, p21^Cip1^, and p53 in mouse embryonic fibroblasts and human IMR90 fibroblasts, in which cellular senescence was induced by oxidative stress and etoposide treatment [24].

Other studies in progeroid mouse models (XFE progeroid syndrome of accelerated aging caused by a defect in DNA repair), revealed that NF-κB activity increased with age and that genetic depletion of the NF-κB p65 subunit delayed the onset of age-related symptoms, reduced inflammatory and degenerative pathology, and decreased oxidative DNA damage, demonstrating that NF-κB activation is correlated with aging but also mechanistically contributed to age-associated inflammation and tissue dysfunction. Pharmacological suppression of IKK/NF-κB activation recapitulated these effects, further supporting NF-κB as a druggable hub in inflammaging [25].

Targeting redox-dependent activation of NF-κB represents an additional mechanistic strategy for low-grade inflammation. In Ehrlich ascites carcinoma model mice, treatment with the mitochondrial antioxidant MitoQ or doxorubicin reduced tumor progression, attenuated mitochondrial ROS production, restored mitochondrial membrane potential, and enhanced PINK1/Parkin-mediated mitophagy, concomitant with the suppression of NF-κB signaling and downregulation of key proliferative and angiogenic markers, including cyclin D1, MMP-1, CD34, and VEGF [26].

Among the most extensively studied suppressors of NF-κB activation are metformin via AMPK and rapamycin, which are mechanistic target of rapamycin (mTOR) inhibitors that indirectly attenuate NF-κB signaling by reducing the SASP [27]. Natural compounds such as curcumin and resveratrol, which act as NF-κB inhibitors via the modulation of mitogen-activated protein kinase (MAPK) signaling and the activation of sirtuin 1 (SIRT1), have been widely evaluated in randomized controlled clinical trials although their effectiveness remains debatable. Curcumin administration for 12 weeks in a randomized, placebo-controlled trial involving patients with CKD undergoing peritoneal dialysis significantly reduced lipid peroxidation (MDA), although no significant changes in inflammatory markers (TNF-α, IL-6), NF-κB-related gene expression, or antioxidant pathways were observed [28]. Resveratrol supplementation for 4 weeks in a randomized, double-blind, placebo-controlled crossover trial in nondialyzed CKD patients also did not significantly modify Nrf2 or NF-κB gene expression in PBMCs, nor did it affect proinflammatory cytokines or antioxidant biomarkers, suggesting limited short-term efficacy on inflammation–oxidative stress pathways [29].

Overall, from a translational perspective, these data support the concept that NF-κB inhibition can function as a senomorphic strategy, reducing the SASP and inflammatory burden without necessarily inducing cell death. However, complete blockade of NF-κB signaling is unlikely to be desirable, given its essential role in immune defense and tissue homeostasis. Therefore, partial and context-dependent modulation, rather than full suppression, may represent a more physiologically relevant approach to targeting NF-κB in aging.

### 4.2. The NLRP3 Inflammasome Is a Central Amplification Hub in Inflammaging

Another critical molecular sensor activated in both immune and parenchymal cells by age-related signals, including alterations in the gut microbiome, mitochondrial dysfunction with increased mitochondrial ROS production, the release of mitochondrial DNA (mtDNA), crystalline structures (e.g., cholesterol crystals, ceramides, monosodium urate, uric acid), lipotoxic metabolites such as oxidized LDL (oxLDL), and impaired autophagy, is the NLRP3 inflammasome, as reviewed by Lee et al. [30].

**Figure 3:**
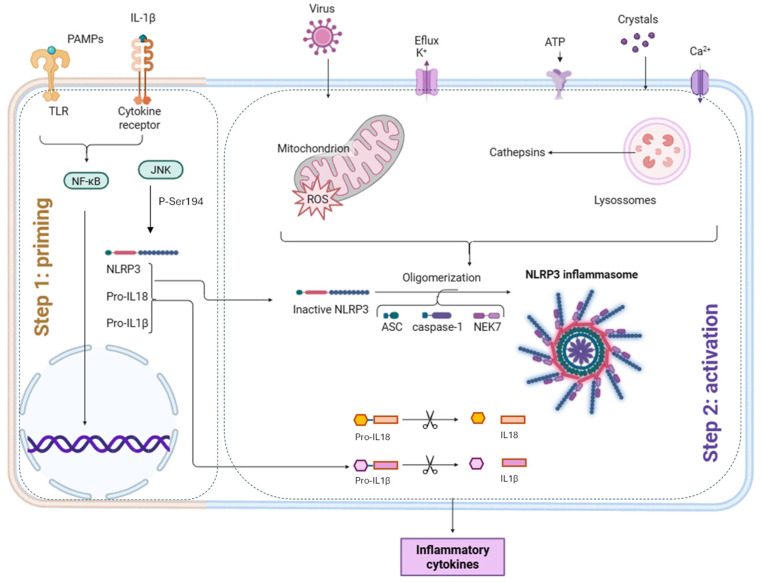
Two-step mechanism of the activation of the NLRP3 inflammasome in immune and parenchymal cells. Step 1: Priming. Pathogen-associated or endogenous inflammatory cues (PAMPs, IL-1β, and TLR ligands) activate NF-κB- and JNK-dependent transcriptional programs, inducing the expression of NLRP3 components and proinflammatory cytokine precursors (pro-IL-1β and pro-IL-18). Step 2: Activation. A second wave of sterile or stress-related danger signals, such as mitochondrial dysfunction and ROS, potassium efflux, ATP signaling, crystalline structures, and lysosomal damage with cathepsin release, promotes conformational activation and oligomerization of NLRP3. Activated NLRP3 recruits the adaptors ASC and procaspase-1 to form a multiprotein inflammasome complex, enabling autocatalytic cleavage of caspase-1. Active caspase-1 subsequently processes pro-IL-1β and pro-IL-18 into their mature bioactive forms, which are secreted and produce local and systemic inflammatory responses. Abbreviations: PAMPs—pathogen-associated molecular patterns, NLRP3—NOD-like receptor family pyrin domain containing 3, TLR—Toll-like receptor, NF-κB—nuclear factor kappa B, JNK—c-Jun N-terminal kinase, ROS—reactive oxygen species, and ASC—apoptosis-associated speck-like protein. Created in BioRender. Tero-Vescan, A. (2026) https://BioRender.com/y4hkrdz (accessed on 10 June 2026).

Once assembled, the NLRP3 inflammasome promotes caspase-1 activation and the subsequent maturation and secretion of the potent proinflammatory cytokines IL-1β and IL-18. These cytokines act both locally and systemically to amplify the inflammatory tone, reinforce NF-κB signaling, and propagate chronic low-grade inflammation characteristic of inflammaging. Importantly, NLRP3 activation is typically submaximal but persistent in aging tissues, making it a key driver of inflammatory amplification rather than an acute pathogen-clearance mechanism [31]. For more mechanistic details, see Figure 3. Studies in progeroid mouse models of accelerated aging revealed increased cardiac and hepatic expression of NLRP3 and caspase-1, whereas pharmacological inhibition of the NLRP3 inflammasome with the selective inhibitor MCC950 improved cellular morphology, reduced inflammasome-dependent inflammation, and significantly extended lifespan, demonstrating a causal and therapeutically targetable role for NLRP3 signaling in age-associated inflammatory dysfunction [32].

In naturally aged C57BL/6J mice, pharmacological inhibition of NLRP3 with the selective inhibitor MCC950 attenuated age-associated metabolic and hepatic dysfunction, accompanied by modulation of phosphoinositide 3-kinase (PI3K)/protein kinase B (AKT)/mTOR signaling, increased autophagic flux, and increased peroxisome proliferator-activated receptor alpha (PPARα) activity. These findings indicate that NLRP3 functions not only as a downstream readout of inflammation, but also as a mechanistic amplifier of metabolic inflammaging that can be therapeutically targeted [21].

Similarly, pharmacological inhibition of the NLRP3 inflammasome with MCC950 in an aging-relevant murine model of heart failure, [induced in mice by a high-fat diet and angiotensin II infusion], significantly reduced systemic IL-18 levels, improved left ventricular function, attenuated cardiomyocyte hypertrophy and fibrosis, and decreased cardiac and visceral adipose tissue macrophage infiltration, indicating that NLRP3 blockade could effectively ameliorate inflammation-driven cardiac and metabolic dysfunction [33].

Metformin has also been shown to attenuate NLRP3 inflammasome activity in clinical contexts such as polycystic ovary syndrome, where a 12-week daily treatment reduced NLRP3 and IL-1β expression and upregulated the expression of anti-inflammatory microRNAs, particularly miR-223. These findings suggest that metformin modulates inflammasome activation not only at the transcriptional level but also through microRNA-mediated posttranscriptional mechanisms, contributing to the attenuation of chronic low-grade inflammation [34].

Targeting upstream or downstream components of the inflammasome pathway represents a complementary therapeutic strategy, as the microtubule inhibitor colchicine has been shown to reduce IL-1β production and systemic inflammation in clinical and experimental settings, while novel derivatives such as a colchicine + myricetin hybrid further suppress NLRP3 inflammasome activation by inhibiting caspase-1, ASC, and GSDMD expression and reducing the expression of proinflammatory cytokines (IL-1β, IL-6, IL-18 and TNF-α) in a rat model of bleomycin-induced lung injury [35]. Additionally, acute IL-1β blockade with anakinra induced rapid immunomodulation in individuals with prediabetes, significantly reducing CRP and leukocyte counts within 12 h, while promoting a shift toward enhanced incretin signaling and early insulin secretion, potentially mediated via IL-6–dependent GLP-1 release [36]. In a neonatal case of multisystem inflammatory disease caused by the NLRP3 mutation p.Gly755Arg, conventional anti-inflammatory therapies were ineffective, whereas targeted IL-1β blockade with canakinumab (2–3 mg/kg every 8 weeks) resulted in rapid resolution of systemic inflammation and sustained clinical remission over 13 months [37].

However, similar to other central inflammatory pathways, complete inhibition of NLRP3 may not be feasible because of its role in host defense and pathogen clearance. Therefore, partial and context-dependent modulation, particularly targeting chronic low-grade activation rather than acute inflammasome responses, may represent a more physiologically appropriate strategy in aging.

### 4.3. cGAS–STING Signaling as a Central Nucleic Acid Sensing Hub in Inflammaging

**Figure 4:**
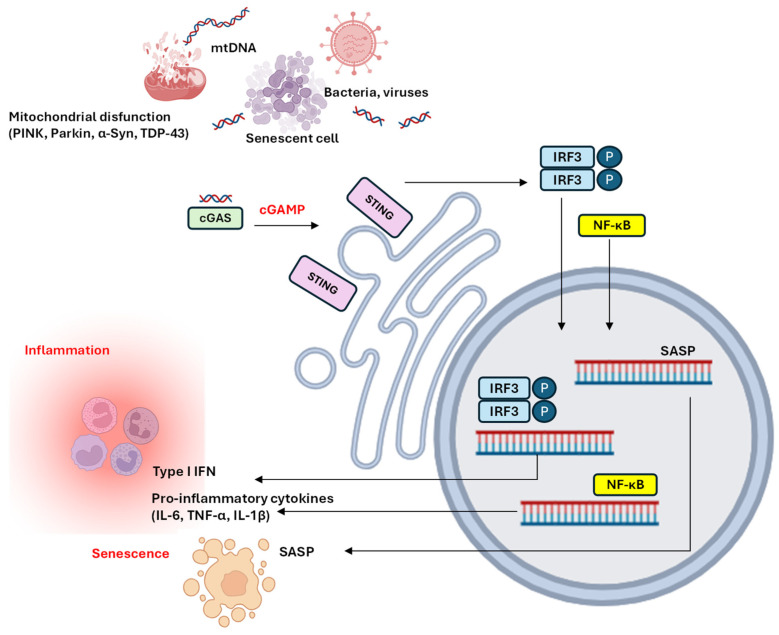
Activation of the cGAS–STING pathway by cytosolic DNA and its contribution to inflammatory and senescence-associated responses. mtDNA released during mitochondrial damage, viral or bacterial DNA, and DNA fragments derived from genomic instability or cellular stress, activate cGAS, which will catalyze the formation of cGAMP, which subsequently binds to and activates the adaptor protein STING located on the endoplasmic reticulum membrane. Activated STING triggers downstream signaling pathways involving the phosphorylation and activation of IRF3 and NF-κB. These transcription factors translocate to the nucleus, where they promote the transcription of genes encoding type I IFNs and proinflammatory cytokines such as IL-6 and TNF-α. Persistent activation of the cGAS–STING signaling axis contributes to chronic inflammation and the induction of SASP. Abbreviations: mtDNA—mitochondrial DNA, cGAS—cyclic GMP–AMP synthase, cGAMP—cyclic GMP–AMP, STING—stimulator of interferon genes, IRF3—interferon regulatory factor 3, NF-κB—nuclear factor kappa B, type I IFNs—type I interferons, IL-6—interleukin-6, TNF-α—tumor necrosis factor-α, SASP—senescence-associated secretory phenotype, and TDP-43—TAR DNA-binding protein 43. Created in BioRender. Tero-Vescan, A. (2026) https://BioRender.com/xal260w (accessed on 10 June 2026).

The cGAS–STING pathway is a cytosolic DNA-sensing axis that detects aberrant accumulation of endogenous DNA in the cytoplasm and converts nucleic-acid stress into innate immune activation. Under conditions of cellular stress associated with aging, including mitochondrial dysfunction, genomic instability, nuclear envelope damage, and defective autophagy, fragments of mitochondrial DNA (mtDNA) or nuclear chromatin can accumulate in the cytosol and activate the DNA sensor cGAS. Activated cGAS catalyzes the synthesis of the second messenger cyclic GMP–AMP (cGAMP), which binds to the adaptor protein STING on the endoplasmic reticulum and triggers downstream signaling through TANK-binding kinase 1 (TBK1) and the transcription factors interferon regulatory factor 3 (IRF3) and NF-κB, ultimately inducing type I interferon responses and proinflammatory gene expression. This pathway therefore provides a direct molecular link between DNA damage, mitochondrial stress, and chronic innate immune activation during aging [38]. For more details, see Figure 4.

Growing experimental evidence indicates that dysregulated activation of the cGAS–STING pathway represents a key driver of age-associated inflammation and tissue dysfunction. In a landmark study, Gulen et al. demonstrated that naturally aged mice exhibit increased activation of cGAS–STING signaling in microglia due to the accumulation of mtDNA released from dysfunctional mitochondria. This aberrant signaling promoted reactive microglial transcriptional states characterized by enhanced type I interferon signaling, ultimately contributing to neurodegeneration and cognitive decline. Importantly, as reviewed eloquently by Gulen et al., H-151-mediated STING inhibition suppressed inflammatory phenotypes in senescent cells, attenuated age-related inflammation across multiple peripheral organs and the brain, and improved tissue function, thereby providing direct experimental evidence that the cGAS–STING pathway acts as a causal driver of inflammaging rather than merely reflecting inflammatory activation [9].

Complementary studies in vascular aging have further reinforced the pathological involvement of cGAS–STING signaling. Increased expression of cGAS, STING, and downstream phosphorylated IRF3 has been detected in experimental models of endothelial senescence, where activation of the pathway promotes endothelial dysfunction and vascular inflammation. Genetic or pharmacological inhibition of cGAS–STING signaling in these models alleviated inflammatory responses and restored vascular homeostasis, suggesting that cytosolic DNA sensing contributes to the development of age-related cardiovascular pathology [39].

In the App^NL-G-F^/hTau double-knock-in mouse model of Alzheimer-like pathology, aberrant activation of the cGAS–STING axis contributes to neurodegenerative processes, whereas concomitant inhibition of STING activity successfully mitigated neuroinflammatory responses and ameliorated disease-related phenotypes. These findings support a central role of DNA-sensing pathways in linking cellular stress to neurodegenerative and inflammatory cascades during aging [40].

Pharmacological targeting of the cGAS–STING pathway might therefore be a promising strategy to attenuate DNA-driven innate immune activation during inflammaging. Among the most widely studied compounds are STING inhibitors, including H-151, C-176, and C-178, which block STING palmitoylation and downstream signaling. In aged mouse models, H-151 effectively suppressed type I interferon responses, reduced systemic inflammation, and improved tissue function, providing strong evidence that STING inhibition could reverse age-associated inflammatory phenotypes [9]. Hydroxychloroquine and chloroquine are broad immunomodulatory and lysosomotropic agents that can attenuate cGAS–STING signaling by interfering with DNA–sensor interactions and nucleic acid sensing. However, unlike selective STING inhibitors such as H-151, C-176, and C-178, they should not be considered specific inhibitors of the cGAS–STING pathway [41]. In parallel, metformin may indirectly suppress cGAS–STING signaling through improving mitochondrial function and reducing mtDNA release, thereby limiting upstream activation of the pathway, as shown in a murine model of colitis, where daily intraperitoneal metformin administration significantly reduced proinflammatory cytokines (TNF-α, IL-6, and IL-1β), decreased epithelial apoptosis, and restored barrier integrity [42].

From a translational perspective, these findings support the concept that cGAS–STING inhibition may function as a senomorphic strategy, reducing chronic interferon signaling and the inflammatory burden without completely abolishing innate immune responses. However, given the essential role of this pathway in antiviral defense, careful dose optimization and context-dependent modulation are critical to avoid immunosuppression.

### 4.4. Low-Grade JAK/STAT Signaling in Inflammaging: Mechanisms and Therapeutic Perspectives

**Figure 5:**
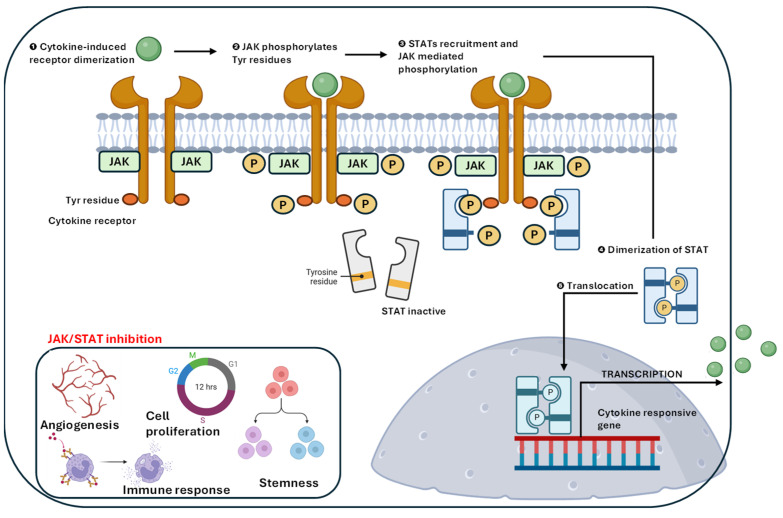
Canonical JAK/STAT signaling pathway activity and its inhibition. Cytokine binding induces receptor dimerization and activation of associated JAKs, which phosphorylate tyrosine residues on the receptor and recruit STAT proteins. Phosphorylated STATs dimerize and translocate to the nucleus, where they regulate the transcription of cytokine-responsive genes involved in inflammation and immune responses. Pharmacological inhibition of JAK/STAT signaling suppresses inflammatory transcriptional programs and can attenuate senescence-associated inflammatory signaling. Abbreviations: JAKs—Janus kinases, STAT—signal transducer and activator of transcription, and P—phosphate. Created in BioRender. Tero-Vescan, A. (2026) https://BioRender.com/9gu4k3f (accessed on 10 June 2026).

The JAK/STAT pathway is an important central hub for cytokine signaling (IL-6 family, interferons and other mediators) that accumulate during chronic low-grade inflammation. In aging tissues, sustained but submaximal JAK/STAT activity promotes the persistence of SASP components, as it is a self-reinforcing circuit, in which cytokine production and STAT activation feed back to maintain a chronic inflammatory tone. This is particularly relevant for STAT3, which integrates signals from IL-6 and related cytokines and has been repeatedly implicated in senescence-associated transcriptional remodeling and age-related pathology (Figure 5).

Inhibition of JAK1/2 in a mouse model of Hutchinson–Gilford progeria syndrome (HGPS), delayed premature aging phenotypes, including reductions in bone fractures, preservation of bone mineral content, improved grip strength, and a trend toward increased survival, providing first in vivo proof-of-principle that sustained dampening of JAK/STAT signaling can modify aging-like inflammatory pathology rather than merely suppress downstream biomarkers [43].

Additional evidence shows that JAK/STAT inhibition does not simply blunt inflammation but can functionally rejuvenate aged progenitor cells. A study in aged tendon-derived stem/progenitor cells showed that aberrant JAK/STAT activation contributed directly to cellular senescence and SASP expression, whereas pharmacological inhibition of the pathway attenuated the expression of senescence markers, reduced the expression of SASP genes, including IL6, IL1B, MMP3, and MMP9, and restored age-impaired self-renewal, migration, stemness, and actin dynamics [44]. TNF-α and IFN-γ synergistically amplify the SASP through a JAK/STAT-dependent signaling, providing experimental evidence that this pathway can integrate inflammatory cytokine networks into senescence-associated transcriptional output. These findings support the concept that low-grade JAK/STAT activation serves as a molecular bridge between chronic cytokine exposure and the maintenance of a pathogenic SASP [45].

Taken together, these studies have established that low-grade JAK/STAT activation is not merely a passive readout of chronic cytokine exposure, but also a mechanistically important and therapeutically actionable persistence hub in inflammaging. By sustaining SASP gene expression, amplifying cytokine-driven feedback loops, and coupling chronic inflammatory signals to tissue degeneration, the JAK/STAT axis represents a plausible target for senomorphic modulation in age-related disease contexts.

Selective JAK1/2 inhibitors such as ruxolitinib and baricitinib attenuate cytokine-driven signaling by preventing the phosphorylation of STAT proteins, particularly STAT3. In parallel, JAK/STAT inhibition disrupts autocrine and paracrine inflammatory loops by lowering IL-6 signaling, thereby limiting pathway self-amplification. Additionally, recent evidence indicates that JAK/STAT blockade may improve mitochondrial homeostasis through reduced ROS production. Overall, suppression of STAT3-dependent signaling contributes to partial restoration of stem/progenitor cell function, including enhanced self-renewal and migratory capacity, as shown in normal human fibroblasts and in a murine model of progeria, suggesting a dual impact on the inflammatory and regenerative aspects of aging [43,44].

Upstream blockade of the IL-6/IL-6R/gp130 axis with tocilizumab (which has been shown to be effective in the treatment of COVID patients) is another potential mechanistic approach, particularly for STAT3-dominant inflammaging phenotypes, as it reduces chromatin accessibility and the transcriptional persistence of SASP components and may attenuate the integration of inflammatory cues with other pathways implicated in inflammaging, including the NF-κB and MAPK signaling pathways [46].

Although numerous experimental data support the use of JAK inhibitors for reducing inflammation and rejuvenating aged progenitor cells, JAK/STAT signaling is fundamentally involved in immune homeostasis, hematopoiesis, and host defense mechanisms. Consequently, its chronic inhibition may increase susceptibility to infections, thromboembolic events, cardiovascular complications, and malignancies. Therefore, a recalibration rather than complete abrogation of this pathway may represent a more appropriate therapeutic strategy.

### 4.5. P38 MAPK Could Be a Stress-Responsive and Druggable Hub in Inflammaging

**Figure 6:**
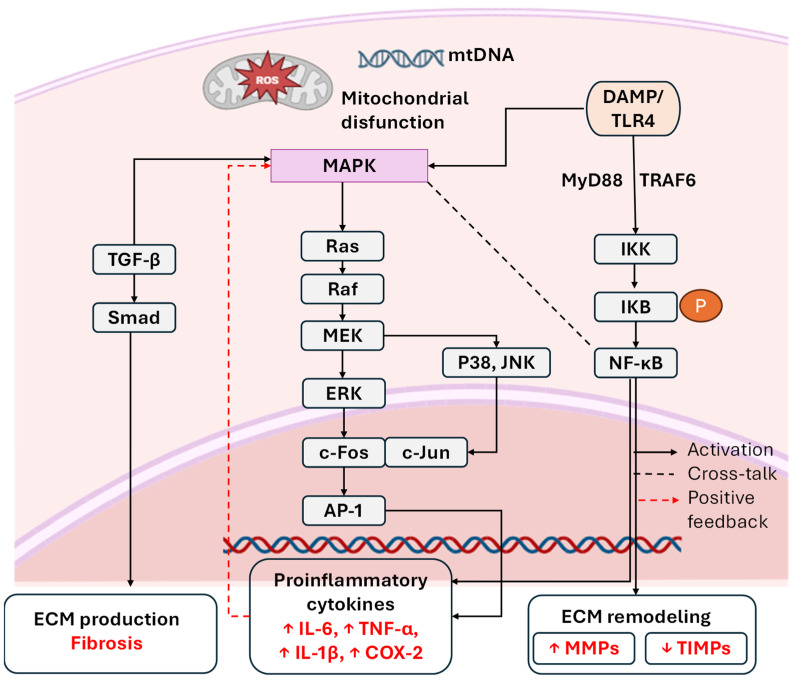
Crosstalk among the MAPK, NF-κB, and TGF-β signaling pathways during inflammation. Mitochondrial dysfunction and DAMP signaling activate MAPK, NF-κB, and TGF-β pathways, which converge to increase proinflammatory cytokines, enhance MMP expression, suppress TIMPs, and promote the chronic inflammation, fibrosis, and tissue remodeling associated with inflammaging. Abbreviations: IL-6—interleukin-6, TNF-α—tumor necrosis factor-alpha, IL-1β—interleukin-1 beta, COX-2—cyclooxygenase-2, TIMPs—tissue inhibitors of metalloproteinases, MMPs—matrix metalloproteinases, MAPK—mitogen-activated protein kinase, NF-κB—nuclear factor kappa B, TGF-β—transforming growth factor-beta, mtDNA—mitochondrial DNA, DAMPs—damage-associated molecular patterns, TLR4—Toll-like receptor 4, MyD88—myeloid differentiation primary response 88, TRAF6—TNF receptor-associated factor 6, ROS—reactive oxygen species, ERK—extracellular signal-regulated kinase, p38—p38 mitogen-activated protein kinase, JNK—c-Jun N-terminal kinase, c-Fos—Fos proto-oncogene, AP-1 transcription factor subunit, c-Jun—Jun proto-oncogene, AP-1 transcription factor subunit, and AP-1—activator protein 1. Created in BioRender. Tero-Vescan, A. (2026) https://BioRender.com/8p02zq3 (accessed on 10 June 2026).

The MAPK p38 signalling pathway is activated by cellular stressors that accumulate during aging, including oxidative stress, inflammatory cytokines (such as TNF-α and IL-1β), mitochondrial dysfunction, and DNA damage. It catalises the phosphorylation of multiple downstream transcriptional regulators, like ATF2, cAMP response element-binding protein (CREB), and NF-κB, promoting the expression of inflammatory genes and reinforcing inflammatory signaling networks. Figure 6 illustrates how mitochondrial dysfunction and mtDNA promote ROS production, which activates MAPK signaling (ERK, p38, and JNK). Concurrently, DAMPs activate TLR4, triggering MyD88/TRAF6-dependent signaling and subsequent IKK-mediated phosphorylation and degradation of IκB, leading to NF-κB activation. MAPK signaling induces c-Fos and c-Jun, which form the AP-1 transcription factor, while NF-κB independently regulates inflammatory gene expression. TGF-β signaling activates Smad-dependent pathways and engages in crosstalk with MAPK signaling, contributing to fibrosis and extracellular matrix remodeling. These pathways converge at the nuclear level to promote the transcription of proinflammatory mediators (IL-6, TNF-α, IL-1β, and COX-2), increase MMPs, and decrease TIMPs, thereby sustaining chronic low-grade inflammation and tissue remodeling characteristics of inflammaging [47]. Importantly, p38 signaling has also been identified as a critical regulator of the SASP, where it contributes to sustained production of proinflammatory cytokines, chemokines, and matrix-remodeling enzymes that propagate chronic inflammatory signaling in aging tissues. Persistent activation of the p38 pathway therefore represents a plausible mechanism linking cellular stress responses to the maintenance of chronic low-grade inflammation characteristic of inflammaging [48].

In a mouse 5XFAD model of neurodegeneration, pharmacological inhibition of p38α MAPK with the selective inhibitor neflamapimod/NJK14047 reduced microglial inflammatory activation, decreased cytokine production, and improved cognitive performance and synaptic integrity, supporting a causal role for p38 signaling in neuroinflammatory neurodegeneration [49]. Further preclinical studies have also shown that pharmacological inhibition of p38α with the same inhibitor reduced Rab5 activity, reversed endosomal pathology, and restored the number and morphology of basal forebrain cholinergic neurons in a mouse model of cholinergic neurodegeneration, providing experimental evidence that p38α signaling contributes to neurodegenerative processes and represents a therapeutically targetable pathway [50].

Similarly, experimental studies in splenic CD4^+^ T lymphocytes isolated from C57BL/6 mice exposed to subcytotoxic ROS demonstrated that activation of p38 MAPK promotes stress-induced cellular senescence, mitochondrial dysfunction, and the production of a proinflammatory Th17-type SASP, whereas pharmacological inhibition of p38 with the selective inhibitor BIRB-796 reversed these senescent phenotypes, highlighting p38 MAPK as a central regulator of immune cell senescence and inflammaging-related inflammatory signaling [51].

Clinical studies have evaluated the selective p38α MAPK inhibitor neflamapimod (VX-745) in patients with neurodegenerative disorders, including Alzheimer’s disease and dementia with Lewy bodies, where treatment reduced biomarkers of synaptic dysfunction and neuroinflammation, supporting the translational potential of p38 inhibition in human inflammatory pathology [52]. The p38 MAPK inhibitor losmapimod has also been evaluated in clinical trials for inflammatory and muscle degenerative disorders, where treatment improved functional outcomes, including muscle strength and mobility, further supporting the clinical importance of targeting p38 signaling [53].

In an in vitro human blood–brain barrier model exposed to ROS, the pharmacological inhibition of p38 MAPK with BIRB-796, together with NF-κB blockade or senolytic treatment, attenuated endothelial cell senescence, reduced proinflammatory cytokine release like IL-8, monocyte chemoattractant protein-1 (MCP-1), and intercellular adhesion molecule-1 (ICAM-1), restored tight junction integrity, and improved barrier function, supporting the role of p38/NF-κB signaling as a key mediator of vascular inflammaging [54]. Pharmacological inhibition of p38α/β MAPK with SKF-86002 in a transgenic mouse model of dementia with Lewy bodies and Parkinson’s disease reduced neuroinflammation, reduced synaptic and neurodegenerative deficits, improved motor function, and promoted the redistribution of p38γ to synapses, while decreasing α-synuclein accumulation, effects that were largely mediated through modulation of microglial activity [55].

In human IMR90 fibroblasts, nicotinamide phosphoribosyltransferase (NAMPT) driven NAD^+^ metabolism was shown to regulate the proinflammatory SASP independently of cell cycle arrest. Mechanistically, the NAMPT–NAD^+^ axis suppressed AMPK activity, thereby relieving p53-mediated inhibition of p38 MAPK, resulting in increased NF-κB activation and sustained inflammatory cytokine production. Notably, these findings suggest that NAD^+^ may be a context-dependent rheostat of inflammation, exerting cytoprotective effects in non-senescent cells while promoting proinflammatory signaling in senescent states [56].

Taken together, the evidence suggests that p38 MAPK is a mechanistically central and therapeutically actionable node linking cellular stress, immune dysregulation, and senescence; therefore, its selective modulation might be a viable strategy to recalibrate, rather than suppress, inflammatory signaling in aging.

The most relevant signaling pathways and their representative modulators are summarized in Table 1.

## 5. Molecular and Metabolic Drivers of Endothelial Inflammaging: From SASP to Therapeutic Targeting

The vascular endothelium is increasingly recognized as a dynamic immunometabolic organ that actively participates in the regulation of inflammatory responses during aging. Under physiological conditions, endothelial cells maintain vascular homeostasis through the balanced production of vasodilatory mediators, particularly nitric oxide (NO), and the suppression of leukocyte adhesion. However, with aging, cumulative exposure to oxidative stress, mitochondrial dysfunction, and chronic low-grade inflammatory stimuli promotes a phenotypic shift toward a proinflammatory and prosenescent state, a process termed endothelial inflammaging.

One of the earliest hallmarks of endothelial dysfunction is the progressive depletion of NO, primarily due to reduced endothelial nitric oxide synthase (eNOS) activity and increased scavenging of NO by ROS. This imbalance leads to impaired vasodilation, increased vascular stiffness, and enhanced susceptibility to thrombosis [60]. In parallel, endothelial cells exhibit increased expression of adhesion molecules such as vascular cell adhesion molecule-1 (VCAM-1) and intercellular adhesion molecule-1 (ICAM-1), which facilitate leukocyte recruitment and transendothelial migration, thereby amplifying local inflammatory responses [61].

At the molecular level, endothelial inflammaging is driven by the activation of redox-sensitive and inflammatory signaling pathways, including the NF-κB, MAPK, and the NLRP3 inflammasome, which converge to sustain the SASP. Senescent endothelial cells exhibit reduced glucose uptake, diminished glycolytic flux, lower lactate production, increased fatty acid oxidation, elevated intracellular ATP levels and increased production of proinflammatory cytokines (IL-6, IL-1β, TNF-α), chemokines (MCP-1), and matrix-degrading enzymes, further promoting vascular remodeling and dysfunction [62]. When human cord blood-derived primary endothelial colony-forming cells undergo senescence via serial culture or stress induced using the DNA modulator Etoposide, they accumulate cytosolic self-RNA concomitant with activation of the retinoic acid-inducible gene I (RIG-I) pathway, leading to enhanced IL-6 and IL-8 secretion. RIG-I silencing reduced inflammation and delayed senescence progression [63]. Similarly, activation of the cGAS–STING pathway has been shown to drive endothelial senescence and vascular inflammation in vivo, particularly in diabetic retinopathy models, as reviewed in [64].

Studies on primary HBMECs stratified by passage (young, presenescent, senescent) revealed that presenescent cells, despite lacking classical senescence markers but exhibiting telomere shortening, developed early bioenergetic defects characterized by reduced glycolysis and impaired mitochondrial respiration, which lowered ATP efficiency and promoted redox imbalance. Ultimately, it was hypothesized that this created a permissive metabolic environment enabling the subsequent activation of proinflammatory pathways (e.g., NF-κB) and the emergence of the SASP in fully senescent endothelial cells [65].

These findings establish a mechanistic framework linking endothelial inflammaging to identifiable biomarker layers, including senescence markers (p16, p21, p53, and SA-β-gal), inflammatory mediators (IL-6, IL-8, IL-1β), endothelial activation markers (ICAM-1, VCAM-1), oxidative stress and DNA damage indicators (ROS, 8-OHdG, γH2AX), and pathway-specific signatures such as interferon-stimulated genes, RIG-I, and cGAS–STING activity, thereby offering potential translational targets for modulating vascular aging.

From a therapeutic perspective, endothelial inflammaging can be targeted through senomorphic and senolytic strategies that act on the central hubs of the SASP and endothelial dysfunction. In vitro studies have shown that metformin reduced doxorubicin-induced endothelial senescence, whilst concomitantly decreasing the secretion of SASP factors and adhesion molecules, and attenuating the LPS-induced hyperinflammatory response via the inhibition of JNK/NF-κB [66]. Similarly, atorvastatin mitigated radiation-induced endothelial dysfunction, PAI-1 secretion, and transendothelial monocyte migration through inhibition of the JNK/c-Jun pathway [67].

At the metabolic level, folic acid has recently been identified as a pharmacological modulator of the glucose-6-phosphate dehydrogenase (G6PD)–NADPH axis, alleviating vascular aging in human aortic endothelial cells by increasing reduced glutathione and inhibiting HDAC3 [68]. In parallel, mTOR inhibition with Rapalink-1 reduced endothelial senescence and the SASP [69], while endothelial-specific genetic suppression of mTOR improved (reduced) arterial stiffness, inflammation, and oxidative stress in **C57BL/6** aged mice [70]. Other promising experimental strategies include inhibition of the NLRP3 inflammasome and modulation of the IL-1β/NLRP3 axis via Ang-(1-7) and klotho, which reduced endothelial senescence and vascular dysfunction in HUVECs [71], as well as targeting the cGAS–STING pathway with RU.521 or H-151, which effectively restored eNOS expression and reduced inflammation and senescence markers in human aortic endothelial cells and aged mouse aorta [39]. Finally, senolytic approaches such as dasatinib plus quercetin or ABT-263 have been demonstrated to enhance vascular regeneration and reduce the functional consequences of senescent endothelial cell accumulation in cerebrovascular models [72], while inhibition of p38 MAPK/NF-κB signaling or senolysis with dasatinib + quercetin protects the blood–brain barrier against oxidative senescence and endothelial SASP [54]. Detailed effects of these compounds on endothelial function are presented in Table 2.

## 6. Adipose Tissue–Immune Crosstalk in Inflammaging and Druggable Immunometabolic Networks

Adipose tissue is an active immunometabolic hub in which adipocytes, macrophages, lymphocytes, endothelial cells, and stromal cells continuously exchange inflammatory and metabolic signals, creating chronic low-grade inflammation, impaired tissue remodeling, and systemic metabolic dysfunction during aging and obesity [73].

In a multiomic mouse study, Cottam et al. reported that obesity shifted adipose macrophage populations toward a more proinflammatory phenotype, with higher macrophage polarization indices and persistent expansion of lipid-associated macrophages even after weight loss, indicating that adipose immune remodeling is not fully reversible and may imprint later inflammaging trajectories. Notably, this inflammatory macrophage state worsened again with weight regain, emphasizing that white adipose tissue (WAT) immune memory contributes to chronic metabolic inflammation rather than simply reflecting fat mass alone [74]. In a mouse model of diet-induced obesity, Feng et al. demonstrated that deletion of the receptor for advanced glycation end products (RAGE) reduced macrophage accumulation in adipose tissue, lowered the expression of M1-polarization genes, improved glucose tolerance, and was associated with increased adipose tissue browning. These findings suggest that the AGE-RAGE axis amplifies WAT inflammation by promoting monocyte recruitment and inflammatory macrophage activation, thereby linking metabolic stress to adipose immune dysfunction [75].

A second major contributor to adipose tissue–immune crosstalk is leptin resistance and hyperleptinemia, which are tightly linked to inflammaging. Leptin enhances the inflammatory effects of LPS in macrophages, increasing cytokine production, glycolytic flux, and mitochondrial remodeling through an mTORC2-dependent mechanism. In vivo, myeloid leptin receptor deletion improved insulin resistance and reduced systemic inflammation in obese mice by improving immunometabolic fitness [76].

WAT is the main site of adipokine secretion in obese individuals; however, brown adipose tissue (BAT) is not immunologically inert, as shown by single-cell transcriptomic analysis and CellChat-based ligand–receptor mapping of adipocytes, progenitors, endothelial cells, Schwann cells, and immune cells, especially during thermogenic remodeling. Cold exposure increased these multicellular interactions in C57BL mice exposed to cold temperatures (5^0^C), indicating that immune and stromovascular crosstalk is an intrinsic component of BAT function, not a secondary phenomenon [77].

Aging disrupts this BAT immune niche in a manner distinct from WAT inflammation. Aged BAT in mice and rats accumulated proinflammatory, senescent S100A8+ immune cells, mainly T cells and neutrophils, which impaired their sympathetic innervation and thermogenic capacity. Xenotransplantation of human S100A8+ immune cells into mice was sufficient to induce aging-like BAT dysfunction, while pharmacologic inhibition of S100A8 with paquinimod rejuvenated BAT axonal networks and thermogenic function. These findings indicated that, whereas WAT inflammaging is dominated by macrophage-rich inflammatory remodeling and adipokine dysregulation, BAT aging is strongly shaped by senescent immune-cell infiltration that compromises neuroimmune control of thermogenesis [78].

From a therapeutic perspective, adipose tissue–immune crosstalk may be targeted through interventions that suppress M1-like macrophage polarization, blunt maladaptive leptin-driven immunometabolic signaling, and preserve BAT immune niche integrity. Metformin attenuates macrophage HIF1α-dependent inflammatory programming and improves brown adipose tissue function in obese mice, whereas myeloid leptin signaling suppression reduces macrophage inflammatory fitness, insulin resistance, and systemic low-grade inflammation, supporting leptin-pathway modulation as a plausible anti-inflammaging strategy [79].

In aged white adipose tissue, senolytic treatment with dasatinib plus quercetin decreases the senescence burden, inflammatory SASP expression, and immune-cell accumulation [80], while NLRP3 inhibition with MCC950 represents a complementary approach for inflammasome-driven metaflammation [81]. Potentially new adipose-targeted strategies include the use of simvastatin-loaded nanoparticles that selectively reprogram inflammatory macrophages and promote local adipose browning [82], as well as S100A8 inhibition with paquinimod, which rejuvenates aged brown adipose tissue by counteracting the deleterious effects of senescent immune-cell infiltration [78]. Together, these findings indicate that pharmacological attenuation of adipose inflammaging will likely require combined senomorphic, senolytic, and immune-metabolic approaches adapted to the distinct biology of white and brown adipose depots.

In addition, metabolic modulators such as trimetazidine and meldonium may represent emerging immunometabolic interventions, as they promote a shift from fatty acid β-oxidation toward glucose utilization through inhibition of key enzymes such as 3-ketoacyl-CoA thiolase and γ-butyrobetaine hydroxylase, respectively, thereby reducing mitochondrial oxidative stress, improving metabolic efficiency, and potentially attenuating proinflammatory macrophage polarization within adipose tissue [83,84].

## 7. Translational Challenges and Future Directions in Inflammaging

### 7.1. The Critical Role of Early-Life Interventions vs. Ethical Considerations

One of the major challenges in geroscience is determining the optimal timing of intervention, as theoretical and experimental evidence suggests that early-life or mid-life interventions may confer disproportionately greater long-term benefits than treatments initiated at advanced stages of aging. However, this concept remains insufficiently explored in humans, largely due to ethical constraints that limit interventional studies in younger, otherwise healthy populations, while the limited translatability of animal data further restricts our ability to define the true preventive window for targeting inflammaging.

Supporting the concept of time-dependent intervention, experimental evidence demonstrates that a brief pulse of rapamycin administered in early adulthood in both *Drosophila* and mice results in lifespan extension and preservation of intestinal function, through sustained activation of autophagy and long-term maintenance of gut barrier integrity, even months after drug withdrawal, similar to the life extension seen in chronically treated organisms [85].

In an in vivo murine model, cellular senescence was induced in adipose-derived preadipocytes by exposure to 10 Gy ionizing radiation (resulting in >85% senescent cells), which were subsequently transplanted intraperitoneally into young (6-month-old) wild-type mice. Notably, even the transfer of relatively small numbers of senescent cells was sufficient to induce persistent physical dysfunction and propagate senescence to host tissues, while intermittent oral administration of the senolytic combination of dasatinib plus quercetin significantly reduced the senescent cell burden, attenuated frailty-associated inflammatory cytokine secretion, and improved survival outcomes [86].

These findings support the fascinating concept that inflammaging is not merely cumulative but may be programmable, with early interventions targeting senescence and immune remodeling yielding long-term benefits.

### 7.2. Chronic Versus Intermittent Therapeutic Strategies

Another major translational challenge is determining whether inflammaging should be treated continuously or intermittently, as chronic suppression of inflammatory pathways may impair immune function, whereas intermittent strategies appear to preserve efficacy while minimizing adverse effects.

The identification of a critical mid-life (13–15 months) decline in Nrf2-dependent neural stem cell function in rats supports the concept that aging occurs within defined temporal windows rather than as a purely linear process. This provides a mechanistic rationale for intermittent, time-targeted therapeutic strategies in inflammaging, where interventions applied during vulnerable transition phases may be more effective than continuous treatment. Notably, the same study showed that the restoration of Nrf2 reversed key aspects of the aging phenotype, indicating that stem cells maintained their functional plasticity and may be therapeutically modifiable, particularly when targeted within a critical temporal window [87].

Another key study (mentioned briefly above) identifying a temporal window for intervention was that of Juricic et al., which demonstrated, in a translational model combining *Drosophila* and mice, that brief rapamycin exposure in early adulthood was sufficient to induce long-lasting geroprotective effects. Specifically, female *Drosophila* and C3B6F1 mice treated with rapamycin between 3 and 6 months of age exhibited lifespan extension and preservation of intestinal function comparable to those of lifelong treatment. These effects are mediated by the sustained activation of autophagy in intestinal enterocytes, suggesting that transient mTOR inhibition can durably reprogram tissue homeostasis [85].

In a controlled murine study, male C3B6F1 mice treated from 6 months of age with dietary rapamycin either continuously or intermittently (1 week on/1 week off) presented the same positive preserved lifespan extension as male animals, whilst partially reducing metabolic side effects such as glucose intolerance. However, continuous treatment still remained more effective in preventing age-related pathologies, indicating a trade-off between efficacy and tolerability in long-term mTOR modulation [88].

In addition, long-term administration of low-dose rapamycin (every 5 days for 6 months, starting at 12 weeks of age) improved mitochondrial respiratory control and ATP production in the liver of senescence-prone (SAMP8) and senescence-resistant (SAMR1) mice. In addition, a reduction in glucose uptake in SAMP8 mice, together with immune regulation through decreased splenic FoxP3^+^ lymphocytes and reduced thymic CD3^+^ cells, was noted. However, despite its metabolic benefits, rapamycin induced measurable immunosuppressive effects, including reduced lymphoproliferative capacity and altered cytokine profiles, highlighting that even low-dose intermittent regimens require careful optimization to balance geroprotective efficacy with immune competence [89].

A recent research perspective supports the existence of a critical developmental window during which mTOR signaling determines the trajectory of aging. Early-life inhibition of mTOR by rapamycin extends lifespan, whereas growth hormone exposure during the same period accelerates aging, highlighting a mirror-like relationship between anabolic signaling and longevity. These findings support the hyperfunction theory, according to which aging represents a continuation of developmental growth programs driven by pathways such as mTOR [90].

### 7.3. Sex-Specific Regulation of Immune Aging and Inflammaging Pathways

Experimental and clinical evidence demonstrate marked sexual dimorphism in inflammatory profiles between males influenced by hormonal, metabolic, and immune regulatory networks.

In a prepubertal Wistar rat model of LPS-induced systemic inflammatory response syndrome (SIRS), males exhibited more severe hepatic and pulmonary injury, whereas females showed reduced tissue damage despite markedly higher circulating endotoxin levels. Both sexes developed systemic immunosuppression, characterized by reduced cytokine production (IL-2, IL-4, TNF-α, TGF-β) and thymic atrophy, but displayed distinct immune remodeling patterns, with decreased CD4^+^ T cells and regulatory T cells in females and reduced B cells in males [91]. These findings suggest that sex-specific inflammatory responses are, at least in part, independent of sex hormones and may be driven by intrinsic genetic and immunoregulatory mechanisms.

Sex hormones modulate not only the magnitude but also the quality of immune responses, influencing long-term inflammatory trajectories and susceptibility to inflammaging. In C57BL/6 mice, estrogen signaling enhances antiviral immunoglobulin isotype switching, with wild-type females exhibiting higher IgG2b levels than estrogen receptor-deficient or ovariectomized mice, while estrogen supplementation restores these responses. Interestingly, testosterone supplementation also increased IgG2b levels, likely through its conversion to estrogen [92].

In murine RAW 264.7 macrophages and primary microglial cultures, pretreatment with 17β-estradiol prior to inflammatory stimulation with LPS or TNF-α inhibited NF-κB nuclear translocation, reduced DNA binding activity, and suppressed the expression of proinflammatory genes, including iNOS and MIP-2. Mechanistically, this effect was mediated via rapid, nongenomic activation of PI3K signaling and cytoplasmic sequestration of NF-κB, independent of IκB degradation [93], together with restored expression of κB-Ras2, a key endogenous inhibitor of NF-κB signaling [94].

Experimental studies suggest that estrogen may exert neuroprotective effects after traumatic brain injury by modulating mitochondrial function, as ex vivo 17β-estradiol treatment altered ROS production, bioenergetics, and oxidative metabolism in mitochondria isolated from injured cortices in a sex-dependent manner, with male mitochondria showing greater susceptibility to trauma-induced dysfunction [95]. Aging-associated proinflammatory markers such as IL-6 and hsCRP have peak values in mid-to-late adulthood. Notably, sex-specific patterns were identified, with both males and females exhibiting elevated inflammatory markers, but women showing stronger correlations between IL-6 and hsCRP with aging, suggesting distinct regulatory dynamics of inflammaging between sexes [96].

In vivo studies in LDL receptor-deficient mice have also shown sex-specific effects on NLRP3 inflammasome activation, with females exhibiting heightened NLRP3 activity, increased IL-1β/IL-18 production, and greater macrophage inflammasome activation compared to males [97]. Sex-dependent differences are also present in metabolic–immune crosstalk within adipose tissue, with experimental studies in murine models of obesity demonstrating that males developed a proinflammatory adipose microenvironment earlier and exhibited increased macrophage infiltration and inflammatory gene expression [98]. This was distinct from females, where increased accumulation and activity of regulatory T cells in adipose tissue contributed to an anti-inflammatory milieu with a distinct immunoregulatory profile [99].

### 7.4. Limitations and Regulatory Challenges: Aging Is Not Classified as a Disease

One of the major challenges in the development of interventional geroscience clinical trials is that aging is not classified as a disease by major regulatory agencies, such as the U.S. Food and Drug Administration and the European Medicines Agency, and, for ethical considerations, such studies are not directly authorized. Consequently, therapeutic strategies are evaluated indirectly through age-related or chronic diseases that mimic inflammatory conditions like those observed during aging. These challenges are further exacerbated by the heterogeneity of aging patterns and the lack of robust, validated biomarkers that accurately reflect biological aging.

Several pioneering attempts, such as the Targeting Aging with Metformin (TAME) trial and the HOME trial, tried to introduce the concept of aging as a modifiable clinical target by using composite endpoints that include multiple age-related diseases and functional outcomes with the main purpose of integrating biological aging markers, epigenetic clocks, inflammatory signatures, and frailty indices [100] but significant limitations remain, particularly regarding the administration of such interventions in clinically healthy younger individuals, where ethical and regulatory constraints are challenging.

In response to these limitations, there is increasing consensus that the successful translation of anti-inflammaging strategies will require the development of novel regulatory paradigms. These include the validation of biomarkers as surrogate endpoints, the adoption of composite clinical outcomes reflecting frailty and resilience, and the potential reclassification of aging-related processes within clinical and regulatory frameworks. Such advances would enable a shift from disease-specific interventions toward targeting the underlying biology of aging, with broad implications for the prevention of multimorbidity and extension of health span.

### 7.5. Translational Perspective and Evidence Hierarchy

Despite the considerable progress achieved in elucidating the molecular mechanisms underlying inflammaging, the concept of “druggable inflammaging” should still be regarded as an emerging translational framework rather than an established therapeutic paradigm. Much of the evidence supporting the pharmacological modulation of NF-κB, NLRP3 inflammasome, cGAS–STING, JAK/STAT, and p38 MAPK signaling derives from preclinical studies conducted in cellular and animal models. Although these findings provide compelling proof-of-concept evidence, their translation into effective and safe clinical interventions remains limited. Important challenges include the heterogeneity of aging populations, tissue-specific inflammatory responses, long-term safety concerns, and the identification of reliable biomarkers capable of monitoring therapeutic responses. Therefore, further well-designed clinical studies are required to validate whether targeting central inflammaging pathways can effectively delay biological aging and improve health outcomes in humans. To provide a clearer perspective on the translational maturity of the therapeutic strategies discussed in this review, Table 3 summarizes the hierarchy of evidence supporting major interventions targeting inflammaging, ranging from in vitro studies and preclinical animal models to human studies and clinical investigations.

## 8. Conclusions

Inflammaging should no longer be considered solely as a biomarker-defined state of chronic low-grade inflammation but rather as a mechanistically organized and therapeutically actionable biological process. Experimental evidence reviewed in this manuscript demonstrates that diverse upstream drivers, including immunosenescence, gut dysbiosis, metabolic dysfunction, mitochondrial stress, and cellular senescence, converge on a limited number of central signaling hubs, notably NF-κB, NLRP3 inflammasome, cGAS–STING, JAK/STAT, and p38 MAPK pathways. Importantly, pharmacological interventions targeting these hubs have shown beneficial effects across multiple disease models, including cardiovascular disease, metabolic disorders, neurodegeneration, frailty, and age-related tissue dysfunction. Comparative evidence suggests that senotherapeutics, inflammasome inhibitors, JAK/STAT modulators, cGAS–STING inhibitors, and metabolic interventions targeting AMPK, mTOR, NAD^+^ metabolism, and mitochondrial homeostasis may each attenuate specific components of the inflammaging network. Rather than acting as isolated therapeutic approaches, these interventions should be viewed as complementary strategies capable of modulating interconnected inflammatory circuits. In this respect, the available literature supports the emerging paradigm of “druggable inflammaging”, in which targeted modulation of central inflammatory mechanisms may delay biological aging, improve tissue resilience, and reduce the burden of age-associated diseases. Nevertheless, the concept of “druggable inflammaging” should currently be regarded as an emerging translational framework, as most supporting evidence derives from preclinical studies and requires further clinical validation before its therapeutic potential can be fully established. Future studies should focus on identifying patient-specific inflammatory signatures and developing precision-based combinatorial interventions capable of simultaneously targeting multiple mechanistic nodes of inflammaging.

## Figures and Tables

**Table 1:** Key Signaling Pathways in Inflammaging: Triggers, Downstream Effects, and Therapeutic Targets.

**NF-κB (IKK complex)**					
**Triggers in aging**	**Downstream effects relevant to inflammaging**			**General Translational Limitations**	
-TLR ligands/PAMPs - cytokines - ROS - DNA damage	-↑ inflammatory transcription -SASP reinforcement - endothelial activation			- Immunosuppression at NF-κB complete inhibition.	
**Ref**	**Compound**	**Model**	**Therapeutic category**	**Druggability**	**Translational limitations**
[24]	SR12343	Ercc1−/Δ Progeroid Murine Model	Senomorphic/anti-inflammatory	↓ Senescence, ↓ SASP, ↓ fibrosis, ↓ metabolic dysfunction,↓ frailty, ↑ healthspan	Evidence limited to progeroid mice and fibroblast models; long-term safety and efficacy in physiological human aging remain unknown.
		Senescent human IMR90 lung fibroblasts		↓ SA-β-gal, ↓ p16INK4a, ↓ p21CIP1, ↓ TNF-α, ↓ MCP-1	
[26]	MitoQ	EAC tumor-in mice	Mitochondria-targeted antioxidant/Geroprotective	↓ mtROS, ↑ PINK1/Parkin-mediated mitophagy, ↓ NF-κB signaling, ↓ angiogenesis, ↓ tumor progression, ↑ apoptosis	Predominantly preclinical oncology evidence; anti-inflammaging benefits in humans require validation.
[28]	Curcumin	CKD patients on peritoneal dialysis/clinical trial	senomorphic/anti-inflammatory/antioxidant	↓ Oxidative stress, ↓ lipid peroxidation, ↓ uremic toxins, potential ↓ inflammaging-related factors	Limited bioavailability and heterogeneous clinical responses; long-term effects on inflammaging remain unclear.
[29]	Resveratrol	Non-dialyzed CKD patients/clinical trial	Senomorphic/antioxidant	No significant effects on Nrf2/NF-κB, inflammation, or antioxidant defenses.	- limited bioavailability and lack of measurable effects on Nrf2/NF-κB signaling in short intervention studies for resveratrol.
	**NLRP3 inflammasome**				
**Triggers in aging**	**Downstream effects relevant to inflammaging**			**General Translational Limitations**	
- ROS - mitochondrial dysfunction - DAMPs - lipotoxicity	-IL-1β/IL-18 maturation -amplification of sterile inflammation			Complete blockade may compromise innate immunity; context-dependent modulation preferred.	
**Ref**	**Compound**	**Model**	**Therapeutic category**	**Druggability**	**Translational limitations**
[57]	NLRP3 genetic suppression (NLRP3−/−)	Male NLRP3 knockout mice (aging model)	Inflammasome-targeting/anti-inflammatory	↑ Lifespan, ↑ metabolic function, ↓ cardiac hypertrophy/fibrosis, ↑ autophagy,↑ NAD^+^/SIRT1, ↓ cardiac aging.	(NLRP3−/−) Genetic deletion is not directly translatable to clinical therapy; pharmacological alternatives are required.
[33]	MCC950	Aged female HFpEF mice	Senomorphic/Inflammasome-targeting/anti-inflammatory	↓ NLRP3 inflammasome activity, ↓ IL-1β/IL-18, ↓ hypertrophy, ↓ fibrosis, ↓ macrophage infiltration, ↑ cardiac function.	Preclinical evidence only; long-term safety and infection risk associated with chronic NLRP3 inhibition remain uncertain.
[58]		Aged female mice (8-month-old C57BL/6J mice treated for 12 weeks) and Nlrp3−/− mice	Senomorphic (NLRP3 inflammasome-targeting)	↑ Ovarian reserve/fertility, ↑ autophagy, ↓ apoptosis, ↓ follicular atresia, ↓ ovarian aging.	Evidence limited to animal models; reproductive and aging-related benefits require human validation.
[34]	Metformin	Obese and non-obese women with PCOS	Senomorphic (anti-inflammatory/inflammasome-modulating)	↑ miR-223, ↓ NLRP3/IL-1β signaling, modulation of inflammasome-related miRNAs, ↑ metabolic and inflammatory status.	Clinical evidence restricted to PCOS populations; applicability to general inflammaging remains uncertain.
[35]	Colchicine-myricetin hybrid (CMyrH)	BEAS-2B human bronchial epithelial cells	Senomorphic/inflammasome-targeting anti-inflammatory	↑ Cell viability, ↓ bleomycin-induced injury, ↓ toxicity (vs. colchicine)	Early-stage preclinical evidence; pharmacokinetics, safety, and efficacy in humans remain unknown.
		Sprague-Dawley rats		↓ Lung injury, ↓ NLRP3-mediated pyroptosis, ↓ IL-1β/IL-6/IL-18/TNF-α, ↑ survival, ↓ toxicity.	
[36]	Anakinra	Randomized double-blind crossover study in adults with obesity-associated prediabetes	Senomorphic/IL-1β pathway inhibitor	↓ Systemic inflammation (CRP, leukocytes), ↓ innate immune activation, ↑ GLP-1, ↑ insulin secretion, ↑ metabolic function.	Chronic IL-1 blockade may increase infection risk; long-term anti-inflammaging benefits require confirmation.
**cGAS–STING**					
**Triggers in aging**	**Downstream effects relevant to inflammaging**			**General Translational Limitations**	
- Cytosolic DNA (nuclear/mtDNA) - chromatin damage - senescence-associated DNA fragments	- Type I IFN program - microglial activation - neurodegeneration - multi-organ inflammation			Mainly preclinical evidence; antiviral immunity concerns.	
**Ref**	**Compound**	**Model**	**Therapeutic category**	**Druggability**	**Translational limitations**
[9]	H-151 (STING inhibitor)	Aged mice/senescent human fibroblasts/human adipose tissue explants	Senomorphic/cGAS-STING pathway inhibitor	↓ SASP, ↓ neuroinflammation, ↑ physical performance ↑ cognitive function, ↑ neuronal survival.	Primarily preclinical evidence; potential interference with antiviral immunity and host defense requires evaluation.
[39]	RU.521 (cGAS inhibitor)	Aged mice/galactose-induced senescent HAECs	Senomorphic/innate immune pathway inhibitor	↑ Vasodilation, ↑ eNOS/NO signaling, ↓ senescence markers (p53/p21/p16), ↓ inflammation, ↓ vascular aging.	Limited to experimental vascular aging models; human safety and efficacy data are lacking.
	H-151 (STING inhibitor)	Galactose-induced senescent HAECs	Senomorphic/STING inhibitor	↑ Endothelial function (eNOS/NO), ↓ inflammation, ↓ senescence markers, ↓ vascular aging.	Evidence restricted to cellular models; long-term vascular and systemic effects remain unknown.
[40]	H-151	STING (TMEM173)	Senomorphic/innate immune pathway inhibitor	↓ Neuroinflammation, ↓ NLRP3, ↓ Aβ/tau pathology, ↓ synaptic degeneration, ↑ cognitive function.	Preclinical neurodegeneration evidence only; clinical efficacy and safety remain unestablished.
[59]	Hydroxychloroquine)/Chloroquine	HEK293T cells transfected with cGAS/STING constructs	Senomorphic (cGAS-STING pathway modulator)	↓ cGAS-STING signaling, ↓ cGAMP, ↓ IFN-β, ↓ DNA-induced innate immune activation.	Evidence for cGAS-STING inhibition is largely mechanistic and preclinical; anti-inflammaging efficacy remains unproven.
[42]	Metformin	DSS-induced colitis in C57BL/6J mice/STAT3ΔIEC mice	Senomorphic/STAT3-targeting metabolic modulator	↓ Intestinal inflammation, ↑ barrier function, ↓ apoptosis, ↑ microbiota balance, ↓ STAT3 signaling.	Findings are limited to intestinal inflammation models; relevance to systemic inflammaging requires further study.
		LPS-stimulated NCM460 human intestinal epithelial cells		↓ STAT3 signaling, ↑ epithelial barrier integrity, ↓ apoptosis, ↑ cell survival, ↓ inflammation.	
**JAK/STAT (low-grade)**					
**Triggers in aging**	**Downstream effects relevant to inflammaging**			**General Translational Limitations**	
- IL-6 family - SASP feedback	- Persistent inflammatory gene programs - senescence reinforcement			Infection and thromboembolic risk	
**Ref**	**Compound**	**Model**	**Therapeutic category**	**Druggability**	**Translational limitations**
[43]	Ruxolitinib	Human MRC5 fibroblasts expressing progerin and HGPS-derived fibroblasts	Senomorphic (JAK1/2 inhibitor)	↓ Senescence markers (SA-β-gal, p21, p16), ↓ IL-8, ↓ FDPS, ↑ nuclear integrity.	Evidence derived from progeroid syndromes; applicability to physiological aging remains uncertain.
		Zmpste24−/− progeroid mice		↑ Bone health, ↑ physical function, ↑ survival, ↓ progeroid features.	Benefits demonstrated in accelerated aging models; long-term immunosuppressive effects require consideration.
[44]	AG490 (JAK/STAT inhibitor)	Aged tendon stem/progenitor cells (TSPCs) Sprague-Dawley rats	Senomorphic (JAK/STAT pathway inhibitor)	↓ Senescence markers, ↓ cell-cycle arrest, ↓ JAK/STAT-mediated aging phenotype.	Experimental inhibitor with limited translational development; no clinical validation available.
[45]	Ruxolitinib	HUVECs exposed to TNF-α + IFN-γ ± IL-6 (cytokine storm-like model)	Senomorphic (JAK1/2 inhibitor)	↓ JAK/STAT activation, ↓ SASP mediators, ↓ ACE2/DPP4 expression, ↑ endothelial regenerative capacity.	Cell culture evidence only; clinical relevance for inflammaging remains to be established.
	Remdesivir		Senomorphic/anti-inflammatory antiviral	↓ JAK/STAT activation, ↓ SASP mediators, ↓ ACE2/DPP4, ↑ endothelial homeostasis.	Anti-inflammatory effects observed in vitro; relevance outside viral infection settings remains uncertain.
**p38 MAPK**					
**Triggers in aging**	**Downstream effects relevant to inflammaging**			**General Translational Limitations**	
- ROS - DNA damage - cytokines - cellular stress	- SASP amplification - inflammatory transcription - tissue stress responses			Pleiotropic signaling pathway; chronic systemic inhibition may impair normal immune and stress-response functions, requiring selective rather than complete inhibition. Evidence is limited to preclinical Alzheimer’s disease models; the ability of NJK14047 to cross an intact blood–brain barrier, its long-term safety, and efficacy in human aging or neurodegenerative diseases remain to be established.	
**Ref**	**Compound**	**Model**	**Therapeutic category**	**Druggability**	**Translational limitations**
[49]	NJK14047	5XFAD Alzheimer’s disease mice	Senomorphic (p38 MAPK inhibitor)	↓ p38 MAPK signaling, ↓ neuroinflammation, ↓ Aβ burden, ↑ microglial phagocytosis, ↑ cognitive function, ↓ neurodegeneration.	Preclinical evidence only; BBB penetration, long-term safety, and clinical efficacy remain unconfirmed.
		BV2 microglial cells/primary mouse microglia,/primary cortical neurons		↓ Neuroinflammation, ↓ microglia-mediated neuronal damage, ↓ apoptosis, ↑ neuronal survival.	
[50]	Neflamapimod (VX-745)	Ts2 Down syndrome/early-onset Alzheimer’s disease mouse model	Senomorphic (p38α MAPK inhibitor)	↓ p38α/Rab5 signaling, ↑ neuronal and synaptic function, ↑ cognition, ↓ neuroinflammation, ↓ age-related neurodegeneration.	Evidence derived from a specific neurodegeneration model; relevance to physiological aging requires validation.
		Dementia with Lewy Bodies (Phase 2a clinical trial)		↑ Clinical outcomes, ↑ mobility, ↑ functional performance, ↑ tolerability.	Primary cognitive endpoint was not achieved; larger randomized trials are needed.
[51]	BIRB-796 (Doramapimod)	senescent CD4^+^ T lymphocytes	Senomorphic (p38 MAPK inhibitor)	↑ Mitophagy, ↓ ROS, ↓ mitochondrial dysfunction, ↓ SASP, ↓ inflammaging phenotype.	Limited to in vitro immune-cell models; no clinical evidence in aging-related disorders.
[53]	Losmapimod	FSHD myotubes; FSHD xenografts	Senomorphic (p38α/β MAPK inhibitor)	↓ DUX4 activity, ↓ downstream target genes, ↓ inflammatory signaling, ↓ degenerative processes.	Effects mainly demonstrated in immature myotubes; predictive value for mature tissues is uncertain.
		Phase I-III FSHD trials		↑ Functional performance, ↓ muscle fat deposition, ↔ DUX4-driven gene expression.	Phase III trial failed to meet primary endpoints despite encouraging earlier findings.

Abbreviations: Nuclear factor kappa B (NF-κB), IκB kinase (IKK), Toll-like receptor ligands (TLR ligands), pathogen-associated molecular patterns (PAMPs), reactive oxygen species (ROS), deoxyribonucleic acid (DNA), senescence-associated secretory phenotype (SASP), NLR family pyrin domain containing 3 (NLRP3), damage-associated molecular patterns (DAMPs), interleukin-1β/interleukin-18 (IL-1β/IL-18), cyclic GMP–AMP synthase–stimulator of interferon genes (cGAS–STING), type I interferons (Type I IFN), stimulator of interferon genes (STING), Janus kinase/signal transducer and activator of transcription (JAK/STAT), interleukin-6 (IL-6), p38 mitogen-activated protein kinase (p38 MAPK), and p38 alpha isoform (p38α), ↑ up arrow means increase, ↓ down arrow means decrease.

**Table 2:** Senotherapeutic interventions targeting endothelial inflammaging and vascular aging.

Ref	Compound	Model	Therapeutic Category	Druggability	Translational Limitations
[39]	RU.521	Mice (12 mo)	Senomorphic/cGAS inhibitor	↑ Endothelial function (eNOS/NO), ↓ endothelial senescence, ↓ vascular inflammation, ↓ cGAS-STING signaling.	Preclinical evidence only.
	H-151	HAECs	Senomorphic (STING inhibitor)	↑ Endothelial function (eNOS/NO), ↓ inflammation, ↓ senescence markers, ↓ vascular aging	Preclinical evidence only; safety and immune-related risks remain uncertain.
[9]		Senescent HFs/human adipose explants/naturally aged mice (26 mo)		↓ Vascular inflammation, ↓ inflammatory cell infiltration, ↓ SASP signaling.	
[72]	Navitoclax (ABT-263)	Aged BALB/c mice	Senolytic	↑ EPC-mediated vascular repair, ↑ cerebrovascular regeneration, ↓ age-related vascular dysfunction.	Preclinical evidence only; hematologic toxicity limits clinical translation.
	Dasatinib + Quercetin				Limited human evidence; long-term safety remains uncertain.
[54]	BIRB796	H_2_O_2_-induced premature senescence in HBMECs (in vitro human BBB model)	Senomorphic (p38MAPK inhibitor)	↑ BBB integrity and angiogenesis, ↓ endothelial senescence, ↓ p38MAPK/NF-κB signaling, ↓ vascular inflammation.	In vitro evidence only; no clinical validation.
	QNZ (EVP4593)			↑ BBB integrity, ↓ endothelial senescence, ↓ vascular inflammation.	Experimental compound; in vivo efficacy and safety remain unproven.
	Dasatinib + Quercetin		Senolytic/Senomorphic	↑ BBB integrity, ↓ endothelial senescence, ↓ vascular inflammation, ↓ MMP-2 activity.	In vitro evidence only; BBB penetration and long-term safety remain uncertain.
[71]	MCC950	IL-1β-infused C57BL/6J mice; HUVECs	Senomorphic (NLRP3 inflammasome inhibitor)	↓ Endothelial senescence, ↓ vascular inflammation (IL-1β), ↑ endothelium-dependent vasorelaxation, ↓ vascular dysfunction.	Preclinical evidence only; safety of chronic NLRP3 inhibition remains uncertain.
	Ang-(1-7)		Senomorphic (RAS/Mas receptor modulator)	↑ Endothelial vasorelaxation, ↑ klotho expression, ↓ NLRP3/IL-1β signaling, ↓ endothelial dysfunction.	Preclinical evidence only; efficacy in human vascular aging remains unclear.
	Recombinant Klotho		Senomorphic (anti-aging protein)	↑ Endothelial function, ↓ NLRP3/IL-1β signaling, ↓ vascular dysfunction.	Experimental therapy; limited clinical evidence.
	Anakinra		Senomorphic (IL-1 receptor antagonist)	↑ Endothelial vasorelaxation, ↓ endothelial senescence, ↓ vascular inflammation.	Potential infection risk; vascular anti-aging efficacy remains uncertain.
[69]	Rapalink-1	Ethanol-treated HUVECs	Senomorphic (mTOR inhibitor)	↓ Endothelial senescence, ↓ vascular inflammation, ↓ NF-κB/MAPK/mTOR signaling, ↓ SASP.	In vitro evidence only; no animal or clinical validation.
[68]	Folic acid	Ang II-induced HAECs,/Ang II-infused mice	Senomorphic/metabolic rejuvenation agent	↑ Endothelial function, ↓ endothelial senescence, ↓ oxidative stress, ↓ vascular inflammation (VCAM-1, IL-1β, IL-6), ↓ vascular fibrosis.	Preclinical evidence only; efficacy in human vascular aging remains unproven.
[67]	Atorvastatin	Irradiated HUVECs	Senomorphic/anti-inflammatory vascular protector	↓ Endothelial senescence, ↓ vascular inflammation (PAI-1, IL-1β, TNF-α, MCP-1), ↓ leukocyte infiltration, ↑ angiogenesis, ↑ endothelial repair.	Preclinical evidence only; no validation in human vascular aging.
[66]	Metformin	Doxorubicin-induced senescent HUVECs/EA.hy926 EC	Senomorphic/SASP suppressor	↓ Endothelial senescence, ↓ vascular inflammation (IL-6, TNF-α, MCP-1, ICAM-1, E-selectin), ↓ NF-κB/JNK signaling, ↓ SASP.	Limited to cell models; no in vivo or clinical validation.

Abbreviations: ↑ up arrow means increase, ↓ down arrow means decrease.

**Table 3:** Translational maturity of therapeutic strategies targeting inflammaging.

Therapeutic Strategy	Compounds	In Vitro Evidence	Animal Disease Models	Naturally Aged Animals	Human Studies/Clinical Evidence	Representative References
NF-κB modulation	Curcumin, resveratrol, SR12343, MitoQ	✓	✓	✓	Limited	[24,26,28,29]
NLRP3 inflammasome inhibition	MCC950, colchicine, colchicine–myricetin hybrid, anakinra, metformin	✓	✓	✓	Emerging	[33,34,35,36,58]
cGAS–STING inhibition	H-151, RU.521, hydroxychloroquine, chloroquine, metformin *	✓	✓	✓	Very limited	[9,39,40,42,58]
JAK/STAT modulation	Ruxolitinib, AG490, Remdesivir	✓	✓	Limited	Available	[43,44,45]
Metabolic interventions	Metformin, Neflamapimod NJK14047, Losmapimod, Atorvastatin, Folic acid, rapamycin, NAD^+^ boosters	✓	✓	✓	Available	[66,67,68]

Abreviations: ✓ confirms the existence of studies. * Metformin may indirectly suppress cGAS–STING signaling through improving mitochondrial function and reducing mtDNA release.

## Data Availability

No new data were created or analyzed in this study. Data sharing is not applicable to this article.

## Abbreviations

The following abbreviations are used in this manuscript:

AKT	Protein kinase B
AMPK	AMP-activated protein kinase
ASC	Apoptosis-associated speck-like protein
BAT	Brown adipose tissue
CD34	Cluster of differentiation 34
CD4^+^ T cells	CD4-positive T lymphocyte
CD8^+^ T cells	CD8-positive T lymphocyte
c-Fos	Fos proto-oncogene, AP-1 transcription factor subunit
cGAMP	Cyclic GMP–AMP
cGAS	Cyclic GMP–AMP synthase
cGAS	Cyclic GMP–AMP synthase,
c-Jun	Jun proto-oncogene, AP-1 transcription factor subunit
COX-2	Cyclooxygenase-2
CREB	cAMP response element-binding protein
DAMPs	damage r-associated molecular patterns
eNOS	Endothelial nitric oxide synthase
ERK	Extracellular signal-regulated kinase
G6PD	Glucose-6-phosphate dehydrogenase
hsCRP	High-sensitivity C-reactive protein
ICAM-1	Intercellular adhesion molecule-1
IKK	Inhibitor of κB kinase
IL-1β	Interleukin-1β
IL-6	Interleukin-6
IRF3	Interferon regulatory factor 3
JAK	Janus kinase
JNK	c-Jun N-terminal kinase
LPS	Lipopolysaccharide
MCP-1	Monocyte chemoattractant protein-1
MHC class II	Major histocompatibility complex class II molecules
MMP-1	Matrix metalloproteinase-1
mtDNA	Mitochondrial DNA
mTOR	Mechanistic target of rapamycin
MyD88	Myeloid differentiation primary response 88
NADPH	Reduced nicotinamide adenine dinucleotide phosphate
NAMPT	Nicotinamide phosphoribosyltransferase
NF-κB	Nuclear factor kappa B
NK	Natural killer cells
NLRP3	NOD-like receptor family pyrin domain-containing 3
NO	Nitric oxide
oxLDL	Oxidized LDL
p38 MAPK	p38 mitogen-activated protein kinase
Parkin	E3 ubiquitin-protein ligase
pDCs	Plasmacytoid dendritic cell
PI3K	Phosphoinositide 3-kinase
PINK1	PTEN-induced kinase 1
PPARα	Peroxisome proliferator-activated receptor alpha
RAGE	Receptor for advanced glycation end products
RIG-I	Retinoic acid-inducible gene I
ROS	Reactive oxygen species
SASP	Senescence-associated secretory phenotype
SIRT1	Sirtuin 1
STAT	Signal transducer and activator of transcription
STING	Stimulator of interferon genes
TAK1	TGF-β–activated kinase
TBK1	TANK-binding kinase 1
TDP-43	TAR DNA-binding protein 43
TGF-β	Transforming growth factor-beta
TIMPs	Tissue inhibitors of metalloproteinases
TLR	Toll-like receptor
TLR4	Toll-like receptor 4
TNF-α	Tumor necrosis factor-α
TRAF6	TNF receptor-associated factor 6
type I IFNs	Type I interferons
VCAM-1	Vascular cell adhesion molecule-1
VEGF	Vascular endothelial growth factor
WAT	White adipose tissue
